# Supplementary material for: Time-Restricted Fasting Improves Liver Steatosis in Non-Alcoholic Fatty Liver Disease—A Single Blinded Crossover Trial
Source: Nutrients. 2023 Nov 22;15(23):4870. doi: 10.3390/nu15234870 (PMC10708421; doi:10.3390/nu15234870)
Supplement: Supplementary file 1 [file nutrients-15-04870-s001.zip › nutrients-2687886-supplementary.pdf]

nutrients

MDPI

Article

Time-Restricted Fasting Improves Liver Steatosis in Non-Alcoholic Fatty Liver Disease—A Single Blinded Crossover Trial

Jack Feehan [1], Alexandra Mack [2], Caroline Tuck [3], Jorge Tchongue [4], Darcy Q. Holt [4], William Sievert [2,4], Gregory T. Moore [2,4] Barbora de Courten [5,*,†] and Alexander Hodge [2,3,4,*,†]

Table S1. Inclusion and exclusion criteria.

Current or prior (past 10 years) alcohol intake ≥30 g/day in men and ≥20 g/day in women.
1. Hepatitis B infection (detectable Hepatitis B surface antigen) or Hepatitis C infection (detectable HCV RNA).
Other liver diseases: autoimmune hepatitis, primary biliary cirrhosis, Wilson's disease, primary sclerosing cholangitis
Poorly controlled Type 2 diabetes mellitus as defined by HbA1C >8.0%
Type 2 diabetes mellitus on insulin, other glucose lowering therapy (GLP-1 agonists, SGLT2- inhibitors and DPP4 inhibitors were not available in the community at the time of this study)
Pregnant or breast-feeding
Cirrhosis with current or a history of decompensated events
Current therapy with:<br>a. Magnesium or aluminium containing antacids<br>b. Anticonvulsants including primidone<br>c. Barbiturates<br>d. Calcitonin<br>e. Bisphosphonates such as: etidronate and pamidronate<br>f. Thiazide diuretics<br>g. Cholestyramine<br>h. Milk thistle<br>i. Vitamin E<br>j. Thiazolidenediones<br>k. Corticosteroids<br>l. Amiodarone<br>m. Tamoxifen<br>n. Methotrexate<br>o. Oestrogen<br>p. Selective serotonin re-uptake inhibitor (started within 4 weeks)
